# Saccharated Calomel

**Published:** 1874-01

**Authors:** 


Honor to Whom Honor is Due.—So valuable do we esteem the Saccharated Calomel as an important addition to our *armamentarium medicorum*, we are glad to do suitable homage to any and all in any way connected with its introduction to our notice. We have received the following communication upon the subject, which we print with pleasure, viz :

*Editor Atlanta Medical and Surgical Journal:*

In the September and October number of the Atlanta Medical and Surgical Journal, page 407, I notice the following :

"Saccharated Calomel.—In our August number, we published a paper upon saccharated calomel, in which the writer was unable to state positively to whom was due the honor of having introduced to the profession this valuable combination. We are happy to be able now to state, upon the authority of the originator himself, that this honor is justly due to Dr. John M. Johnson, of Atlanta, Ga."

In a book, entitled "Selections from Private Prescriptions of Living American Practitioners, by Horace Green, M.D., LL.D. New York, 1858," page 77, I find the following: "As a gentle and more certain laxative and alterative, and one equally well adapted for infancy and childhood, some eminent American practitioners, prefer, altogether, the *Chloride of Mercury* minutely subdivided by being triturated with a large amount of sugar. To one part of calomel add ten parts, by weight of white sugar.

℞.—Hydrag. Chlorid. Mitis, . . . . . ʒj.

Sachari Albi, . . . . . . . ʒx.

Triturate the two substances in a wedgewood mortar for ten or fifteen minutes, so as to subdivide minutely, and intimately to blend the calomel and sugar.

"Those who have never tried the mercury thus prepared, will be surprised at the increased activity which will be imparted to the medicine, by this fine subdivision of its particles: five grains of the above preparation will contain half a grain of calomel, and this small amount, taken on going to bed, will produce, with most persons, a mild laxative effect the following day. With young children, half this amount, or from two to three grains of the medicine placed on the tongue (and children take it as readily as they will pure sugar) will be sufficient, ordinarily, to prove cathartic." D. B. S.

*Colaparchee, Ga., January* 7, 1873.

